# Low Density Lipoproteins Promote Unstable Calcium Handling Accompanied by Reduced SERCA2 and Connexin-40 Expression in Cardiomyocytes

**DOI:** 10.1371/journal.pone.0058128

**Published:** 2013-03-13

**Authors:** Montserrat Barriga, Roi Cal, Nuria Cabello, Anna Llach, Alexander Vallmitjana, Raúl Benítez, Lina Badimon, Juan Cinca, Vicenta Llorente-Cortés, Leif Hove-Madsen

**Affiliations:** 1 Cardiovascular Research Center, Consejo Superior de Investigaciones Cientificas, Insitut Català de Ciencies Cardiovasculars and Institut d'Investigació Biomedica Sant Pau, Barcelona, Spain; 2 Cardiology Service, Institut d'Investigació Biomedica Sant Pau and Universitat Autònoma de Barcelona, Barcelona, Spain; 3 Centro de Investigación Biomédica en Red de Obesidad y Nutricion, Barcelona, Spain; 4 Dept. Ingeniería de Sistemas, Automática e Informática Industrial, Universitat Politècnica de Catalunya, Barcelona, Spain; Tohoku University, Japan

## Abstract

The damaging effects of high plasma levels of cholesterol in the cardiovascular system are widely known, but little attention has been paid to direct effects on cardiomyocyte function. We therefore aimed at testing the hypothesis that Low Density Lipoprotein (LDL) cholesterol affects calcium dynamics and signal propagation in cultured atrial myocytes. For this purpose, mRNA and protein expression levels were determined by real time PCR and western blot analysis, respectively, and intracellular calcium was visualized in fluo-4 loaded atrial HL-1 myocyte cultures subjected to field stimulation. At low stimulation frequencies all cultures had uniform calcium transients at all tested LDL concentrations. However, 500 µg LDL/mL maximally reduced the calcium transient amplitude by 43% from 0.30±0.04 to 0.17±0.02 (p<0.05). Moreover, LDL-cholesterol dose-dependently increased the fraction of alternating and irregular beat-to-beat responses observed when the stimulation interval was shortened. This effect was linked to a concurrent reduction in SERCA2, RyR2, IP3RI and IP3RII mRNA levels. SERCA2 protein levels were also reduced by 43% at 200 µg LDL/mL (p<0.05) and SR calcium loading was reduced by 38±6% (p<0.001). By contrast, HDL-cholesterol had no significant effect on SERCA expression or SR calcium loading. LDL-cholesterol also slowed the conduction velocity of the calcium signal from 3.2+0.2 mm/s without LDL to 1.7±0.1 mm/s with 500 µg LDL/mL (p<0.05). This coincided with a reduction in Cx40 expression (by 44±3%; p<0.05 for mRNA and by 79±2%; p<0.05 for Cx40 protein at 200 µg/ml LDL) whereas the Cx-43 expression did not significantly change. In conclusion, LDL-cholesterol destabilizes calcium handling in cultured atrial myocytes subjected to rapid pacing by reducing SERCA2 and Cx40 expression and by slowing the conduction velocity of the calcium signal.

## Introduction

The damaging effects of hypercholesterolemia in the cardiovascular system are widely known, but little attention has been paid to direct effects on cardiomyocyte function even though most of the adult patients suffering from dyslipemia in industrialized societies are at risk of suffering sudden cardiac death (SDC) caused by arrhythmias[1]. Therefore, an antiarrhythmic potential of cholesterol-lowering drugs may result from either a direct electrophysiological antiarrhythmic effect of these drugs or from an indirect antiarrhythmic action resulting from lowering the cholesterol levels provided that cholesterol have arrhythmogenic actions. Since cardiac arrhythmias among others have been linked to changes in the activity of ion channels[2], [3], [4], altered intracellular calcium handling[2], [5], [6], [7], [8], or disturbances in the conduction of the electrical signal through cardiac gap junctions[9], the antiarrhythmic effects of cholesterol-lowering drugs could be due to a direct or indirect action on one or several of these mechanisms.

Regarding the direct actions of cholesterol-lowering drugs it has been reported that statins can reduce the density of the sacolemmal Na+–K+ pump[10], desensitize beta-adrenergic signalling[11] and reduce beta-adrenergic receptor mediated RAC-1 activation and apoptosis[12], affect the activity of Ca^2+^-activated K^+^ channels in porcine coronary artery smooth muscle cells[13], the expression of genes that regulate calcium homeostasis in skeletal muscle[14], and calcium uptake in smooth muscle cells[15]. Although several of these properties of statins may confer antiarrhythmic activity to statins they have not been directly associated with specific antiarrhythmic actions.

On the other hand, hypercholesterolemia has been associated with electrical remodelling and increased vulnerability to ventricular fibrillation in a rabbit hypercholesterolemic model[16]. Recently, we also reported that very low-density lipoproteins (VLDL) uptake induces intracellular lipid accumulation in cardiomyocytes, which is associated with disturbances in intracellular calcium handling linked to SERCA2 downregulation[17]. These results suggest that lipoprotein-derived intracellular lipids may modulate intracellular calcium handling. Furthermore, hypercholesterolemia has been associated with down-regulation of connexin-40 (Cx40) and connexin-43 (Cx43)[18], [19] and statins have been shown to reverse this effect[18]. Thus, it is conceivable that low density lipoprotein (LDL) uptake and derived intracellular lipid accumulation have direct effects on intracellular calcium homeostasis and signal propagation in cardiac myocytes. To test this hypothesis, we here investigated how exogenous LDL affected cholesterol accumulation in cultured cardiomyocytes and the concurrent effects on calcium dynamics, signal propagation, as well as SERCA2 and connexin expression.

## Methods

### HL-1 cardiomyocyte cell culture

The murine HL-1 cell line was generated by Dr. W.C. Claycomb (Louisiana State University Medical Centre, New Orleans, Louisiana, USA)[3] and kindly provided by Dr. U Rauch (Charité-Universitätmedizin Berlin). These cells showed cardiac characteristics similar to those of adult cardiomyocytes such as the presence of highly ordered myofibrils and cardiac-specific junctions in the form of intercalated disks as well as the presence of cardio-specific voltage dependent currents such as the I_Kr_ and an ultrastructure similar to primary cultures of adult atrial cardiac myocytes[20], [21]. The HL-1 cells were maintained in a Claycomb Medium (JRH Biosciences, Lenexa, KS, USA) supplemented with 10% fetal bovine serum (FBS) (Invitrogen Corporation, Carlsbad, CA, USA), 100 µM norepinephrine, 100 units/mL penicillin, 100 µg/mL streptomycin, and L-Glutamine 2 mM (Sigma Chemical Company, St. Louis, MO, USA) in plastic dishes, coated with 12.5 µg/mL fibronectin and 0.02% gelatin, in a 5% CO_2_ atmosphere at 37°C.

### Lipoprotein isolation and characterization

Human LDLs (d_1.019_–d_1.063_ g/mL) and HDLs (d_1.063_–d_1.210_ g/mL) were obtained from pooled sera of normocholesterolemic anonymous volunteers that provided written informed consent to use the serum for this study. LDL and HDL preparations were less than 24 hours old, non-oxidized (less than 1.2 mmol malonaldehyde/mg protein LDL) and without detectable levels of endotoxin (Limulus Amebocyte Lysate test, Bio Whittaker). The purity of LDLs and HDLs was assessed by agarose gel electrophoresis (Paragon System, Beckmann) and the composition of LDL and HDL was, as expected, cholesterol:protein (2∶1) and (1∶5), respectively. The study was approved by the institutional ethics committee at Hospital of Santa Creu i Sant Pau and conducted in accordance with the Declaration of Helsinki.

### Lipid extraction

At the end of lipoprotein-exposure period, cells were exhaustively washed, twice with PBS, twice with PBS–1% BSA, and twice with PBS–1% BSA–heparin 100 U/mL before they were harvested for intracellular lipid determination or calcium-handling studies as previously described[17], [22]. One aliquot of the cell suspension was extracted with methanol/dichlorometane (2∶1, vol/vol). After solvent removal under an N_2_ stream, the lipid extract was redissolved in dichloromethane and one aliquot was partitioned by thin layer chromatography.

### Determination of cholesteryl ester and free cholesterol intracellular content

Thin layer chromatography (TLC) was performed on silica G-24 plates. The different concentrations of standards were applied to each plate**.** The chromatographic developing solution was heptane/diethylether/acetic acid (74∶21∶4, vol/vol/vol). The spots corresponding to cholesteryl ester (CE) and free cholesterol (FC) were quantified by densitometry against the standard curve of cholesterol palmitate, triglycerides and cholesterol, respectively, using a computing densitometer (Molecular Dynamics).

### Determination of intracellular calcium handling

**Figure 1:**
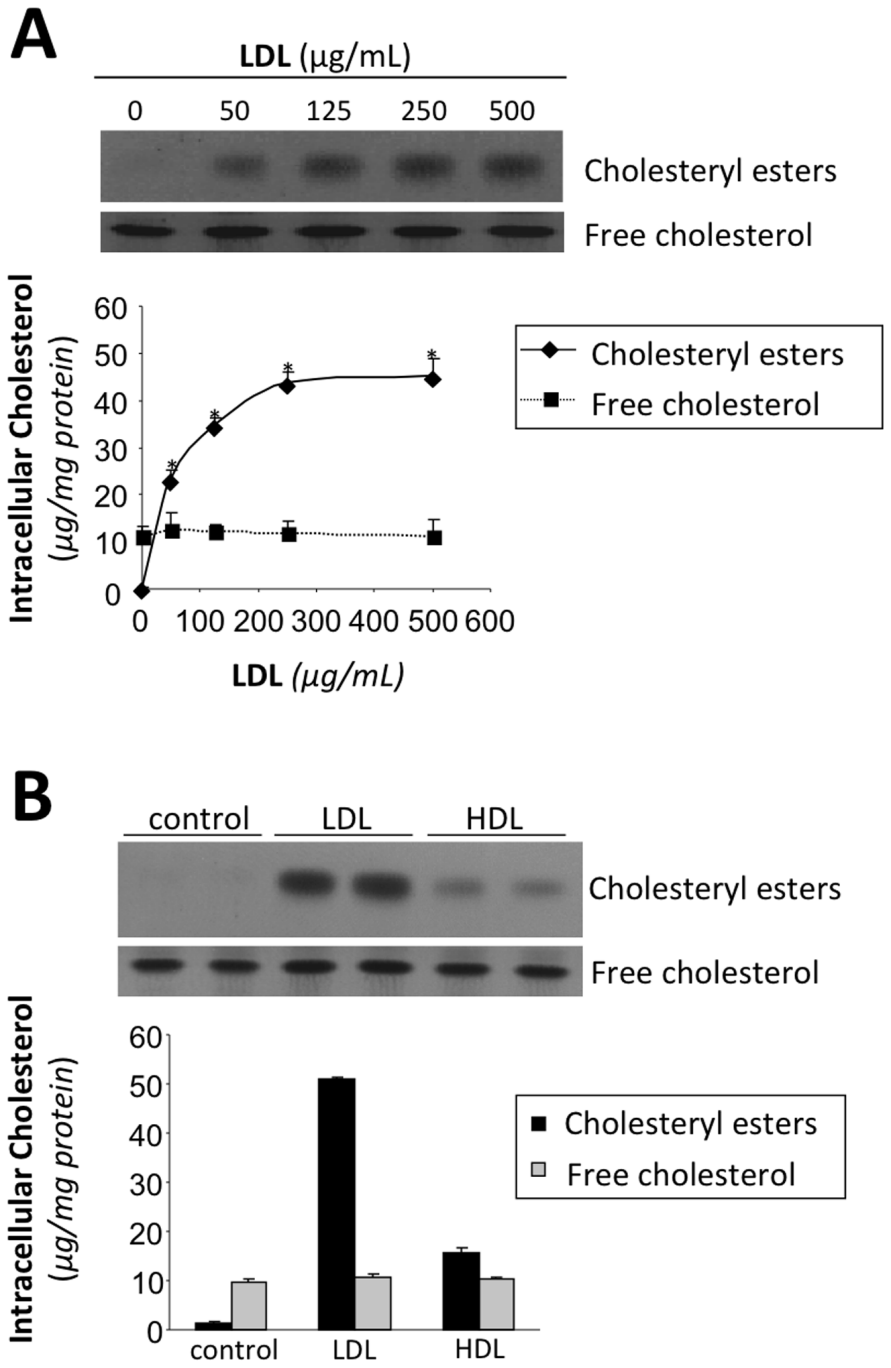
LDL induces intracellular cholesteryl ester accumulation. **A** Thin layer chromatography showing intracellular cholesteryl ester and free cholesterol in HL-1 cell cultures incubated with 0, 50, 125, 250, and 500 µg/mL LDL for 24 hours. Line graphs below show the relationship between the extracellular LDL concentration and intracellular cholesteryl ester (diamonds) and free cholesterol (squares) levels. **B** Thin layer chromatography showing intracellular cholesteryl ester and free cholesterol in HL-1 cell cultures incubated with LDL or HDL (200 µg/mL). Bar graphs below show intracellular cholesteryl ester (black bars) and free cholesterol (gray bars) levels. Results were expressed as microgram of cholesterol per milligram of protein and shown as the mean of three experiments performed in duplicate. Values significantly different from the level with 0 µg/ml LDL are indicated with asterisks.

Twenty-four hours before experimentation, HL-1 cardiomyocytes were incubated in a culture medium containing one of four LDL concentrations: 0. 125, 250 or 500 µg/mL, and the stability and propagation of the calcium signal was recorded upon a step-wise reduction of the stimulation interval from 2.5 to 0.75 s. In some experiments, the effect of LDL and HDL was compared. To detect changes in intracellular calcium handling, cultures were loaded with fluo-4 and calcium was assessed in a 1×1 mm square of the cell culture using a resonance scanning confocal microscope (Leica SP5 AOBS) with a 10x objective (see **figure 1A**). Cell cultures were loaded with 2.5 µM fluo-4AM for 15 min at room temperature followed by 30 min of deesterification. Fluo-4 was excited at 488 nm with the laser intensity set to 20% and subsequently attenuated to 10%. Fluorescence emission was collected between 505 and 650 nm at a frame rate of 25 Hz, and changes in intracellular calcium were estimated by normalizing the fluorescence emission (F) to the fluorescence emission at rest (F_0_). The experimental solution contained (in mM): NaCl 136, KCl 4, NaH_2_PO_4_ 0.33, NaHCO_3_ 4, CaCl_2_ 2, MgCl_2_ 1.6, HEPES 10, Glucose 5, pyruvic acid 5, (pH = 7.4). Calcium signals were detected and characterized off-line using Leica LAS software or a custom made program that automatically detects the calcium signal (baseline fluorescence, peak fluorescence, calcium transient amplitude, and calcium transient duration at half maximum (FDHM) for each cell in the culture. Baseline fluorescence was determined as the lowest 5% of the fluorescent signal at a given stimulation frequency. Values for baseline and peak fluorescence were expressed as the increase above baseline fluorescence in the absence of field stimulation. The calcium transient amplitude was calculated as the difference between the peak fluorescence and the baseline fluorescence immediately before the peak.

To visualize the propagation pattern of the calcium transient through the image field, cell segmentation was performed by inspecting spatial regions presenting large variability in the fluorescence signal. Regions with sizes between 150 and 1500 µm^2^ were identified as cells and their average calcium signal was measured. The delay in the propagation of the calcium transient across the image field was then colour coded for each cell detected.

The propagation pattern of the calcium transient across the image field was also characterized as uniform, alternating or irregular. In this case, the propagation pattern was considered uniform if all calcium transients propagated from the proximal region (next to the stimulation electrode) to the distant region of the image field. Alternating propagation patterns were those showing an alternating response at the distal region, with the extreme being a response on every second pulse at the distant region. Irregular propagation patterns included all responses where the response at the distal region was irregular. The propagation velocity was calculated as the distance between a region of interest proximal and distal to the stimulation electrodes (dX) divided by the time delay between the calcium transients (dt) measured at half maximum.

To estimate SR calcium loading and the activity of the Na^+^–Ca^2+^ exchanger, field stimulation was interrupted and cell cultures were transiently exposed to 10 mM caffeine for 30 s in order to release the calcium stored in the SR. The SR calcium content was estimated from the time integral of the global calcium transient elicited by caffeine. Moreover, tau values obtained by fitting the decay of the calcium transient with an exponential function were used as a measure of the Na^+^–Ca^2+^ exchange activity.

### Real time PCR

Gene expression of SERCA2, Cx40, and Cx43 was assessed by real time PCR-7000 Sequence Detection System of ABIPRISM (Applied Biosystems) using the following assays on demand: mouse Cx40 (Mn01264990_m1), mouse Cx43 (Mn00439105_m1), SERCA2 (Rn01499537_m1), RyR2 (Rn01470303_m1), IP3R-I (Rn01425720_m1), IP3R-III (Rn01470303_m1). Rat ribosomal protein, large, P0 *(ARBP)* (Rn00821065_g1) was used as endogenous control. Taqman real-time PCR was performed with 1-µL/well of RT products (1 µg total RNA) in 10 µL of TaqMan PCR Master Mix (PE Biosystem) with the primers at 300 nM and the probe at 200 nM. PCR was performed at 95°C for 10 minutes (for AmpliTaq Gold activation) and then run for 40 cycles at 95°C for 15 seconds and 60°C for 1 minute on the ABIPRISM 7000 Detection System. The threshold cycle (Ct) values were determined and normalized to endogenous control [17]. Relative quantification using standard curves was used to analyze relative gene expression.

### Western blot

Proteins were analyzed by Western blot analysis as previously described. Blots were incubated with monoclonal antibodies against SERCA2 (Novus Biologicals NB100–237), Cx40 (Invitrogen, 36–4900) and Cx43 (Sigma-Aldrich C6219). Equal loading of protein in each lane was verified staining filters with Pounceau and also by incubating blots with monoclonal antibodies against beta tubulin for HL-1 cardiomyocytes (Abcam, ab6046, dilution 1∶500).

### Statistical analysis

Data were expressed as mean ± SEM. A statview (Abacus Concepts) statistical package for the Macintosh Computer System was used for all analysis. Statistical significance was assessed by ANOVA for repeated measurements and by t-test when appropriate. *P*<0.05 was considered significant.

## Results

### Elevation of exogenous LDL levels induces cholesterol accumulation

To test how exogenous LDL levels affected cholesterol accumulation in cultured cardiomyocyte, HL-1 cardiomyocytes were exposed to increasing concentrations of LDL (0, 50, 125, 250 and 500 µg/mL). As shown in **Figure 1A**, increasing LDL doses increased basal intracellular CE from 2.6±0.2 at baseline to 23.5±1.6 with 50 µg/mL LDL and 45±4 µg CE/mg cell protein with 500 µg/mL LDL. In contrast, HDL (200 µg/mL) slightly increased intracellular CE content of HL-1 cardiomyocytes (**Figure 1B**). As expected, intracellular free cholesterol (FC) levels were not altered by LDL or HDL in HL-1 cardiomyocytes.

### Effect of LDL on the calcium transient

**Figure 2:**
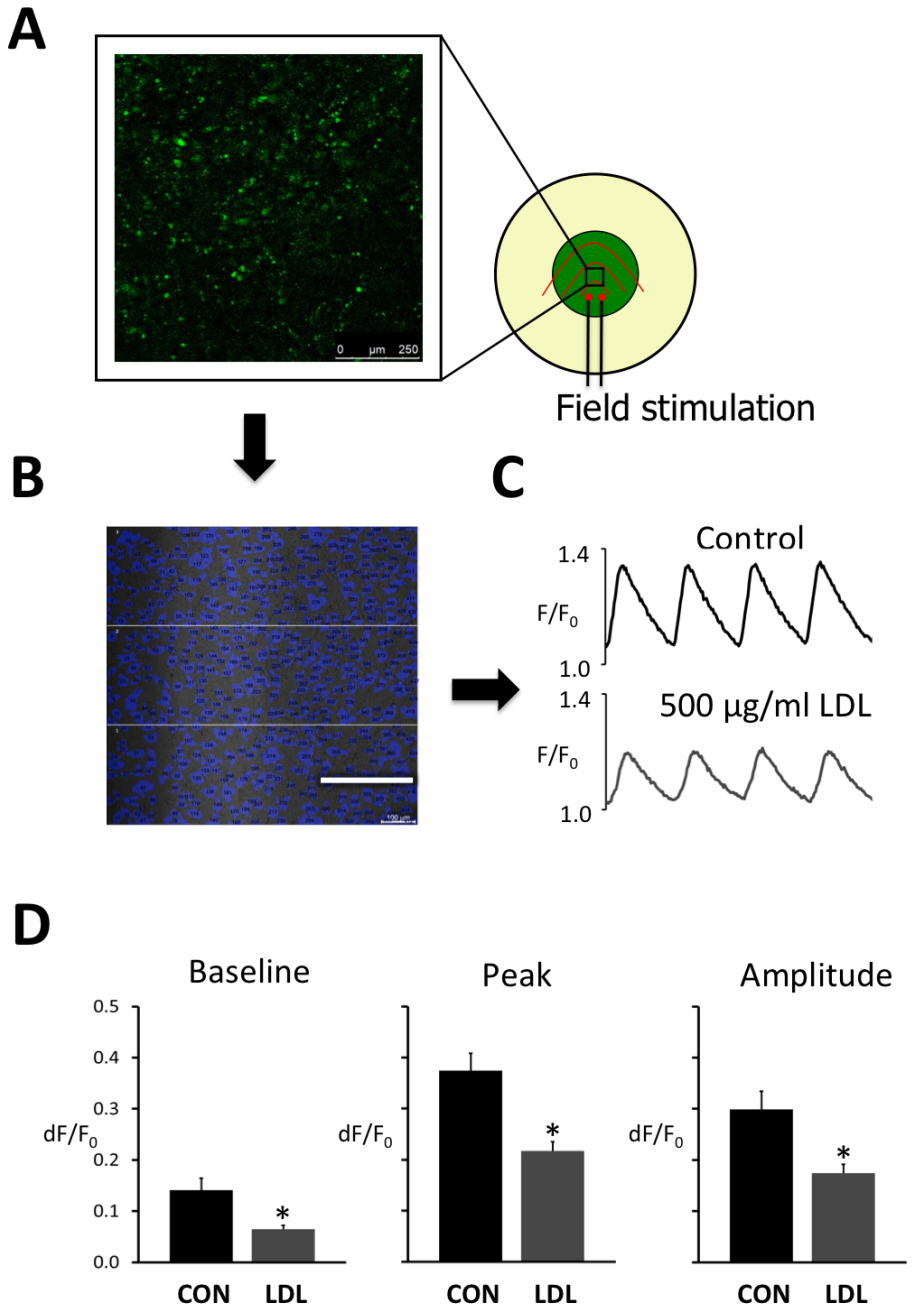
LDL reduces the intracellular Ca^2+^- transient. **A** Schematic representation of a cell culture dish subjected to field stimulation with indication of the 1×1 mm field where calcium imaging is performed. **B** Image of the HL-1 cardiomyocyte culture with individual myocytes showing detectable calcium transients represented in blue. Scale bar is 250 µm. **C** Global calcium transients elicited by field stimulation at 2 s intervals in the absence of LDL (control) and the presence of 500 µg/ml LDL. **D** Average baseline (left panel), peak (center panel) and amplitude (right panel) of the calcium transients recorded without (CON) and with 500 µg/ml LDL. Values were recorded in three different fields from eight HL-1 preparations. Statistical differences between CON and LDL are indicated with an asterisk.

To test if intracellular CE accumulation was associated with changes in intracellular calcium handling, we first examined how exogenous LDL affected the intracellular calcium transient elicited by field stimulation. **Figure 2A** shows schematic outline of a 1×1 mm field of a HL-1 culture subjected to field stimulation and panel 2B shows the image field with indication of 427 myocytes that had calcium transients when subjected to field stimulation. Representative traces of global calcium transients recorded in control conditions and with 500 µg/mL LDL, which induces maximal CE accumulation, are shown in **Figure 2C**. When stimulated at intervals of 2 s, 500 µg/mL LDL reduced the amplitude of the calcium transient by 43% (from 0.30±0.04 to 0.17±0.02, p<0.05), and there was a concurrent reduction in both baseline and peak fluorescence (**Figure 2D**). By contrast, the duration of the calcium transient at half maximum was unaffected by LDL (938±33 ms with LDL vs. 1021±32 ms in control).

**Figure 3:**
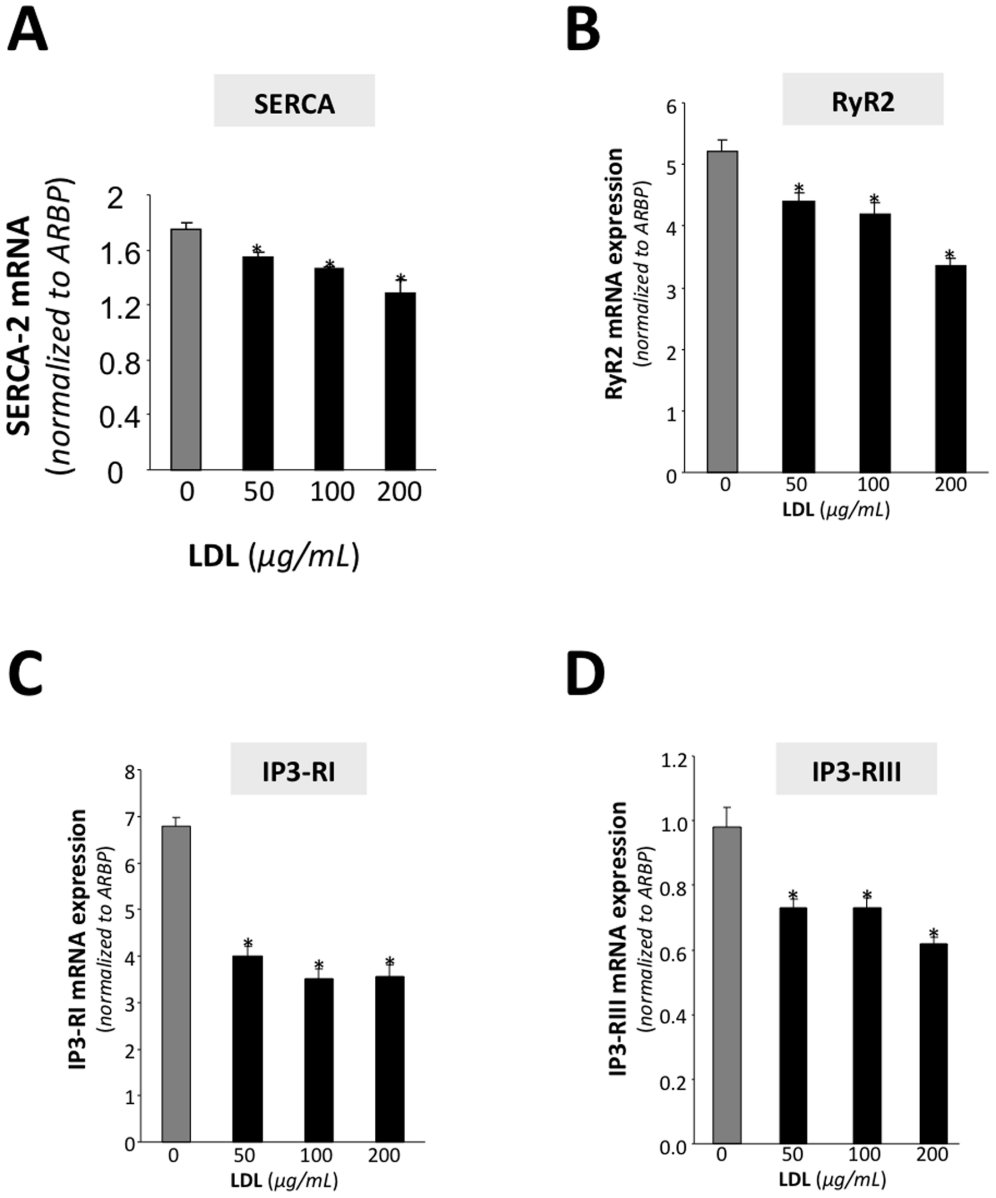
LDL reduces the expression of calcium handling proteins. Real-time PCR analysis showing SERCA2 (A), RyR2 (B), IP3-RI (C) and IP3-RIII mRNA expression levels in HL-1 cardiomyocytes exposed to increasing doses of LDL. Data were processed with a specially designed software programme based on Ct value of each sample and normalized to *ARBP* mRNA. Values significantly different from the level with 0 µg/ml LDL are indicated with asterisks.

**Figure 4:**
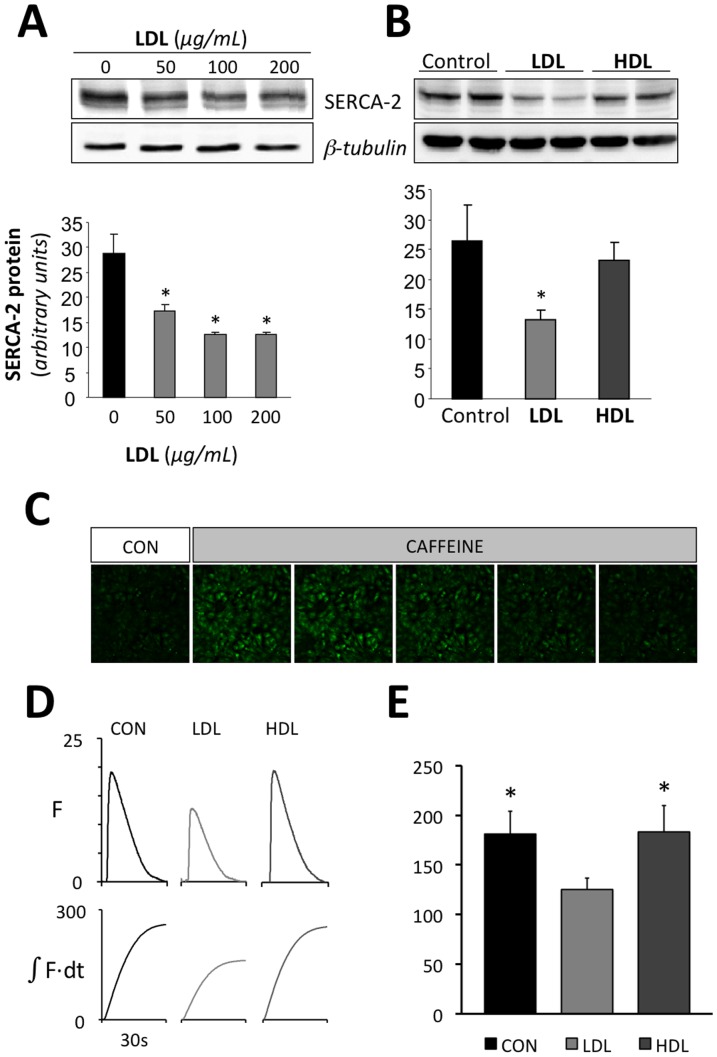
LDL reduces SERCA expression and SR calcium loading. Representative Western blot analysis showing SERCA2 bands in HL-1 cells exposed to increasing LDL concentrations (0, 50, 100 and 200 µg/mL) (**A**) or to similar dose (500 µg/ml) of LDL or HDL (**B**) for 24 hours. The bar graph below shows SERCA2 band quantification. Unchanged levels of β-tubulin are shown as loading control. Results are mean±SEM of three independent experiments performed in duplicate. *P<0.05 versus. HL-1 cells incubated in absence of LDL. **C** Calcium images acquired at rest before (CON) and during a rapid caffeine application. **D** Representative calcium transients recorded in cultures incubated without lipoproteins (CON), with 500 µg/ml LDL or with 500 µg/ml HDL. The lower panel shows the corresponding time integrals of the calcium transients. **E** summary of the effect of LDL and HDL on the time integral of the caffeine induced calcium transient. *P<0.05 vs. cells incubated with LDL

To determine whether the observed changes in the calcium transient were associated to LDL-mediated changes in calcium handling proteins, the mRNA levels of SERCA2, RyR2, IP3-RI and IP3-RII were analyzed at increasing LDL doses. **Figure 3** shows that increasing LDL levels caused a progressive and significant decrease in the mRNA levels of all four calcium handling proteins. Further investigation of the effect of LDL on SERCA levels and SR calcium loading showed that LDL dose-dependently reduced the expression of SERCA2 protein levels (**Figure 4A**). At a concentration of 200 µg/mL LDL, SERCA2 protein was maximally reduced to 43% of the level detected in myocytes incubated without LDL. Importantly, HDL at the same dose, did not exert any significant effect on SERCA-2 protein expression (**Figure 4B**) supporting the notion that the effects of LDL are linked to intracellular lipid accumulation rather than to a non-specific effect on membrane properties. Accordingly, the time integral of the calcium transient elicited by a rapid caffeine application, which releases the SR calcium content, was significantly smaller in cells incubated with 500 µg/mL LDL than in controls or in cells incubated with 500 µg/mL HDL (**Figure 4C–E**). This suggests that LDL but not HDL significantly reduces steady-state SR calcium loading. Moreover, there were no significant differences in the decay of the caffeine induced calcium transient among CON, LDL and HDL treatments (exponential fits yielded tau values of 6.4±0.7, 5.4±0.6 and 6.8±1.2 s respectively), suggesting that LDL treatment does not modify the activity of the Na^+^–Ca^2+^ exchanger.

### Effect of LDL on the stability and propagation of the calcium transient

**Figure 5:**
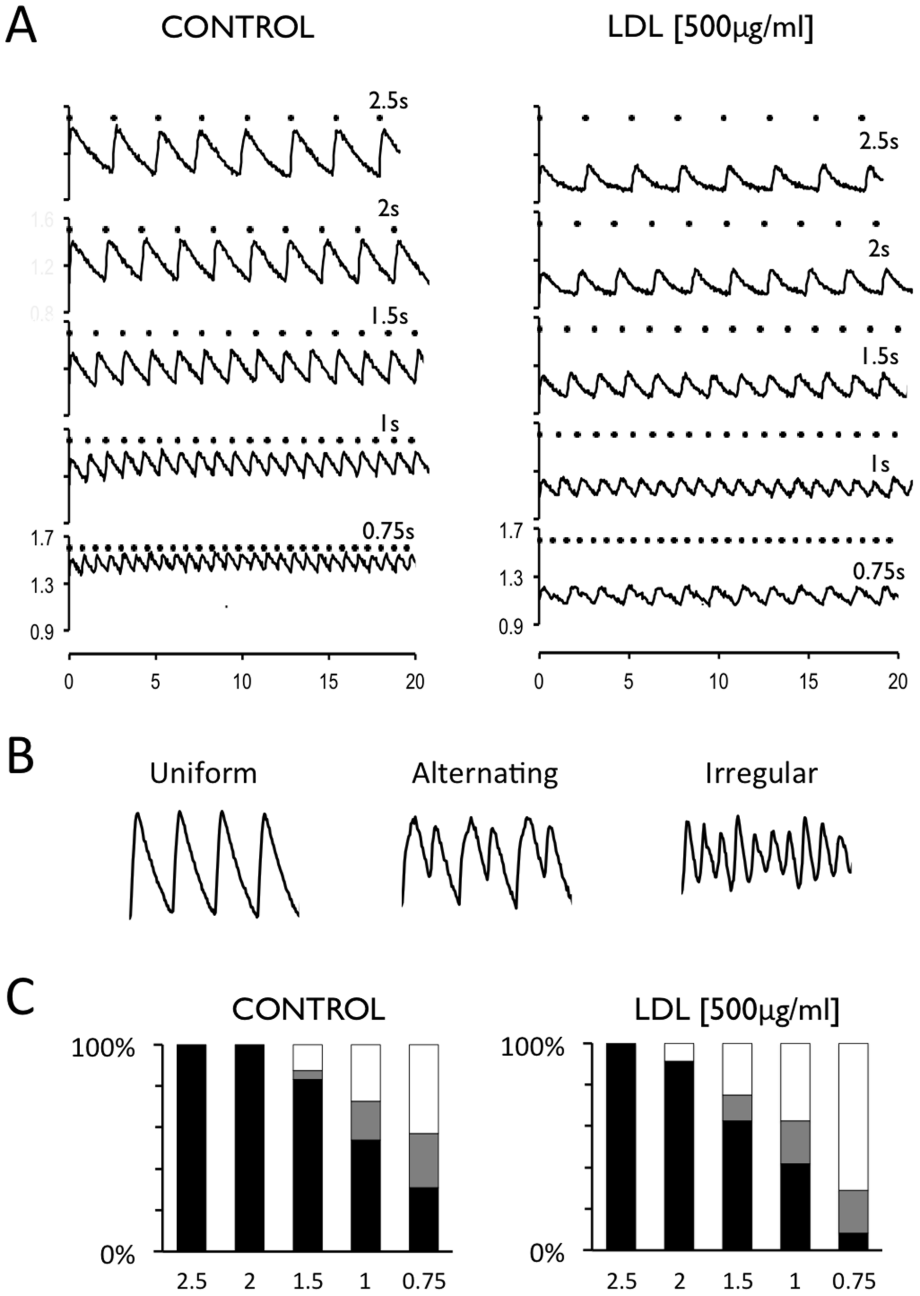
LDL promotes non-uniform Ca^2+^ - transients at short stimulation intervals. **A** Calcium transients recorded at stimulation intervals of 2.5, 2, 1.5, 1 and 0.75 s (top to bottom) in the absence (CONTROL) or the presence of 500 µg/ml LDL (right panel)**.** Each stimulation pulse is indicated with a dot above the traces. **B** Representative traces illustrating uniform, alternating and irregular response. **C** Distribution of uniform (black bar), alternating (grey bar) or irregular (white bar) calcium transient amplitudes at decreasing stimulation intervals recorded in the absence (CONTROL) or the presence of 500 µg/ml LDL (right panel)**.** Values were recorded in four different fields from each of five HL-1 preparations.

To test if the reduction in calcium handling proteins also affected the beat-to-beat stability of the calcium transient, myocyte cultures were subjected to field stimulation at increasingly shorter pacing intervals. **Figure 5A** shows calcium transients elicited at increasingly shorter stimulation intervals in the absence of LDL (Control) and in the presence of 500 µg/mL LDL. The calcium transient amplitudes were uniform at all LDL concentrations with long intervals between stimulation pulses. As the stimulation interval was shortened, cultures started to present alternating (cyclical oscillations between two or three values) or irregular calcium transients (cultures responded in an irregular manner to the stimulation pulses). **Figure 5B** shows examples of uniform, alternating and irregular responses. **Figure 5C** shows the relationship between the stimulation interval and the percentage of preparations presenting uniform, alternating and irregular responses. Moreover, it shows that LDL-cholesterol increased the fraction of irregular responses and decreased the fraction of uniform responses at shorter stimulation intervals. Thus, when stimulated at 0.75 s intervals, the fraction of irregular responses increased from 41% at 0 µg/ml LDL to 71% at 500 µg/mL LDL and the fraction of uniform responses decreased from 30% to 8%.

**Figure 6:**
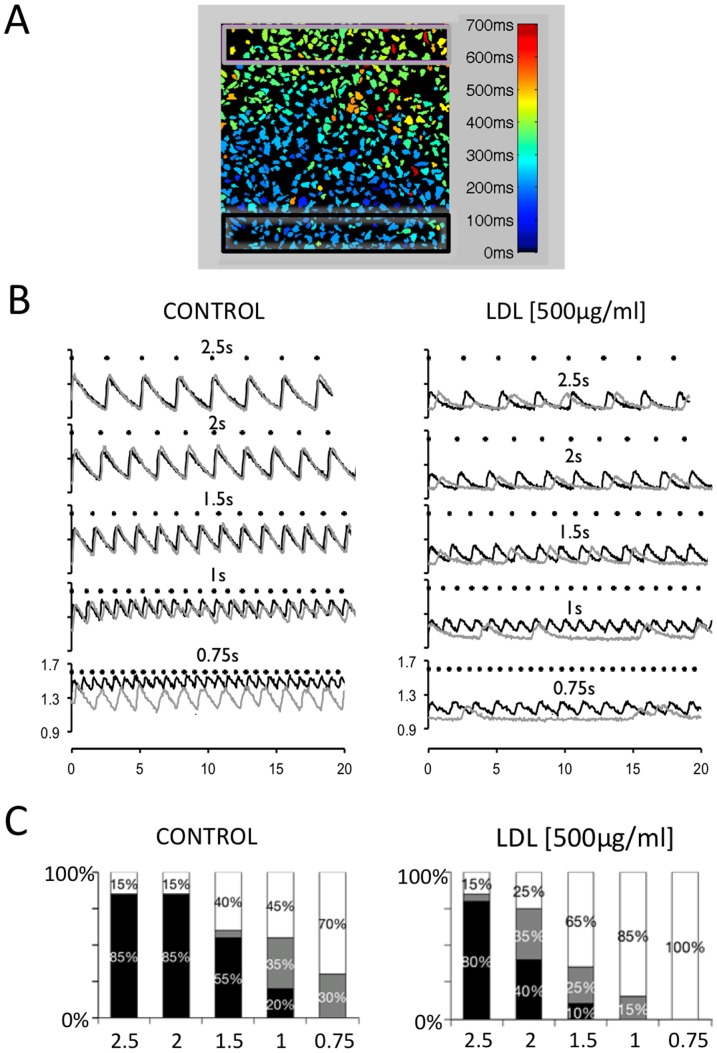
LDL prevents uniform propagation of the Ca^2+^ - transient. **A** HL-1 image field with the activation time for individual myocytes shown in color code. Black and light grey rectangles at the bottom and the top of the images indicate regions used to measure calcium transients proximal and distal to the stimulation electrodes respectively. Image field is 1×1 mm. **B** Calcium transients recorded in the black and grey rectangles are shown in black and grey traces. Recordings were performed at stimulation intervals of 2.5, 2, 1.5, 1, and 0.75 s (top to bottom) in the absence of LDL (CONTROL) or the presence of 500 µg/ml LDL (right panel). Notice that LDL disrupted a uniform propagation of the calcium transient from the dark to the light grey rectangle. **C** Rate dependent distribution of the propagation pattern recorded with 0 (CONTROL) and 500 µg/ml LDL (indicated above figures). The stimulation interval is given below each bar (in seconds) and the propagation patterns were classified as uniform (black bars), alternating (grey bars) or irregular (white bars). See methods for details. Values were recorded in four different fields from each of five HL-1 preparations.

To determine if exogenous LDL affected the propagation of the calcium signal across the myocyte culture, calcium transients were measured in two regions of interest proximal and distant to the stimulation electrodes. **Figure 6A** shows a map of an image field with indication of the activation time for each myocyte in the field. The black rectangle indicates the region used to quantify the calcium transient proximal to the stimulation electrode and the calcium transient distant to the stimulation electrodes was quantified in the region delimited by the light grey rectangle. **Figure 6B** illustrates how incubation with LDL dramatically impaired the ability of calcium signal to propagate uniformly from the proximal to the distant region. **Figure 6C** shows how LDL dose-dependently increased the fraction of non-uniform propagation patterns.

**Figure 7:**
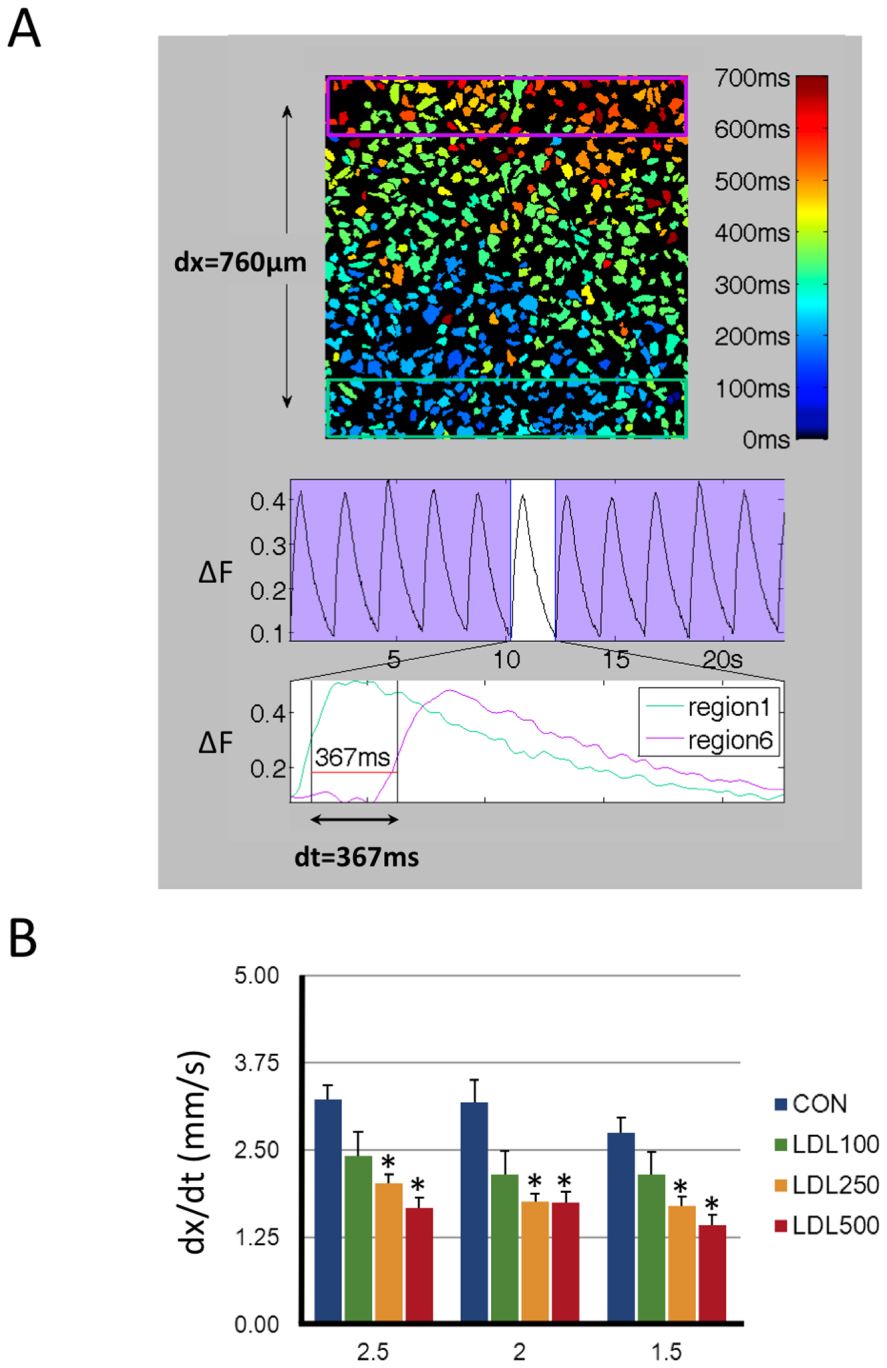
LDL slows the propagation velocity of the Ca^2+^ - transient. **A** HL-1 image field with the activation time for individual myocytes shown in color code. Green and pink rectangles at the bottom and the top of the images indicate regions used to measure calcium transients proximal and distal to the stimulation electrodes respectively. Image field is 1×1 mm. The middle panel shows global calcium transients and the lower panes shows local calcium transients recorded in green and pink rectangles. The propagation velocity (dx/dt) was calculated as distance between the two rectangles divided by the time elapsed between the green and pink calcium transients at their half maximum (dt, indicated below traces). **B** Effect of 0 (Control), 100, 250, and 500 µg/ml LDL on the propagation velocity (dx/dt) recorded at stimulation intervals of 2.5, 2, and 1.5 s. Values were recorded in four different fields from each of five HL-1 preparations. Values statistically different from the corresponding Control value are indicated with asterisks.

To determine if the promotion of non-uniform propagation patterns by increasing LDL-doses was linked to changes in the propagation velocity, this parameter was determined as the time required for the calcium transient to advance from the proximal (green rectangle) to the distant (pink rectangle) region of the myocyte culture as shown in **Figure 7A**. **Figure 7B** shows that LDL-dose dependently reduced the propagation velocity (dx/dt) at stimulation frequencies where signal propagation was uniform.

### Effect of LDL on connexin-40 and connexin-43 expression

**Figure 8:**
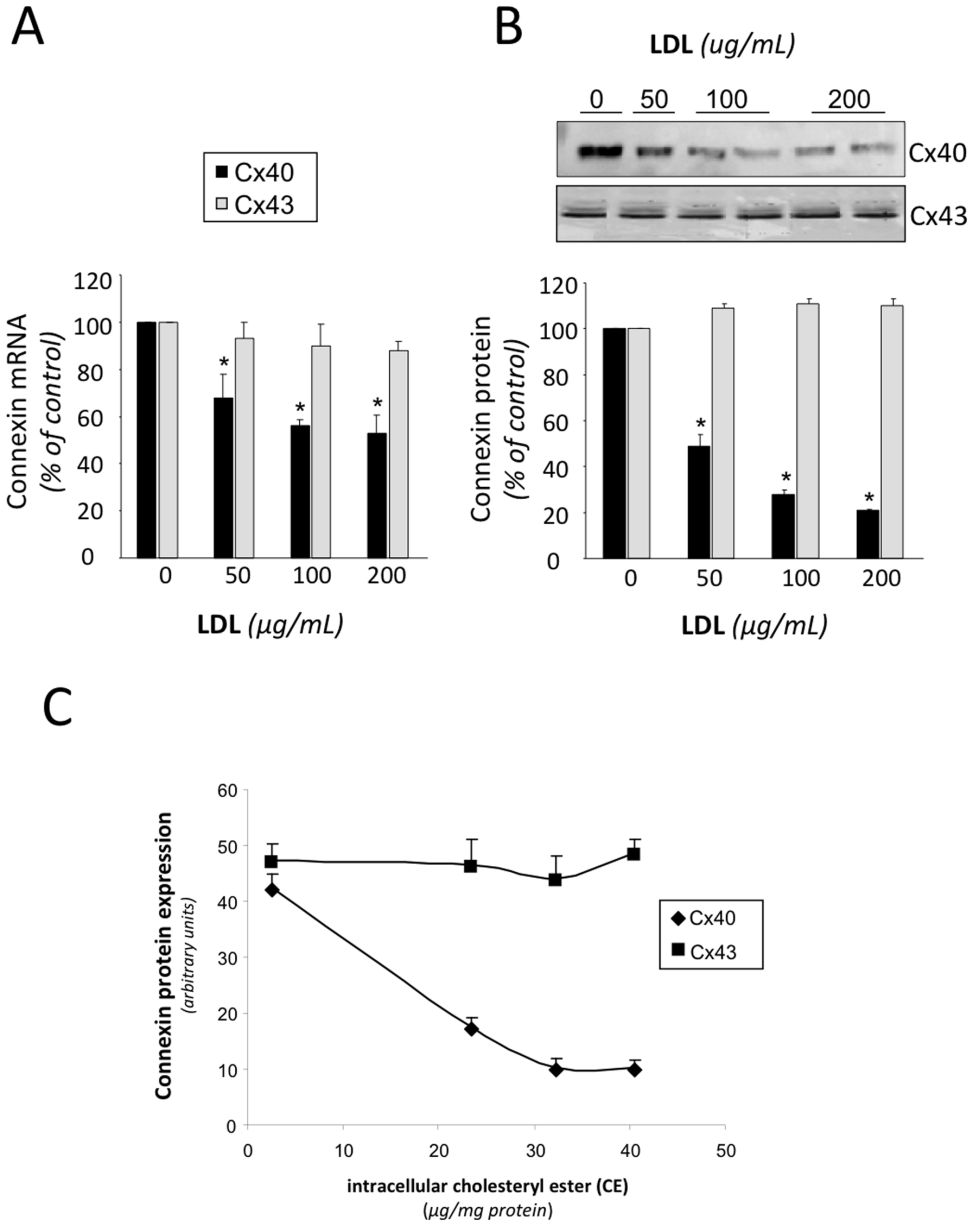
LDL reduces connexin-40 expression in HL-1 myocytes. Effect of increasing doses of LDL (0, 50, 100 and 200 µg/mL) on connexin-40 (Cx40) and connexin-43 (Cx43) mRNA (**A**) or protein (**B**) levels in HL-1 myocyte cultures **C** Relationship between intracellular cholesteryl ester (CE) and Cx40 and Cx43 protein levels in HL-1 myocyte cultures. Results are shown as mean±SEM of three independent experiments performed in duplicate. *P<0.05 vs. cells incubated in absence of LDL.

To determine whether alterations in the propagation of the calcium signal were associated to changes in the expression of connexins, we analyzed the effect of increasing dose of LDL on Cx40 and Cx43 expression in HL-1 cardiomyocytes. LDL at 200 µg/mL maximally reduced Cx40 mRNA expression by 45% (**Figure 8A**) and Cx40 protein expression by 79% (**Figure 8B**). In contrast, LDL did not exert a significant effect on Cx43 mRNA or protein expression. Interestingly, we found an inverse correlation between intracellular CE content and Cx40 protein expression (**Figure 8C**) in HL-1 cardiomyocytes.

## Discussion

The main finding of this study is that LDL-cholesterol reduces the calcium transient in a dose-dependent manner, destabilizes calcium handling on a beat-to-beat basis and slows the conduction velocity of the calcium signal in cultured atrial myocytes subjected to rapid pacing. This alteration of calcium handling is accompanied by an LDL-dependent down-regulation of Cx40, SERCA2 and other calcium-handling proteins.

### LDL-mediated reduction of the calcium transient

We have recently shown that exposure of cultured cardiomyocytes to high VLDL doses induce intracellular CE and triglyceride accumulation and a concurrent reduction in SERCA2 expression, SR calcium loading, and in the intracellular calcium transient[17]. The present data show that exposure to pathological doses of LDL also promotes CE accumulation, reduces SERCA2, RyR2, IP3-RI and IP3-RII expression, lowers SR calcium loading, and diminishes the calcium transient; supporting the notion that intracellular CE accumulation impairs SR calcium handling. In agreement with this, our results show that HDL only induced a slight intracellular cholesteryl ester accumulation in HL-1 cardiomyocytes and had no significant effect on SERCA-2 expression or SR calcium loading. In fact, a previous study performed in a hypercholesterolemic animal model showed that SERCA2 expression inversely correlated with CE lipid enrichment of the sarcoplasmic reticulum[23].

### LDL-mediated destabilization of the beat-to-beat response

Interestingly, high extracellular LDL levels also altered the beat-to-beat stability of the calcium transient when myocyte cultures were subjected to increased stimulation frequency. Theoretically, the LDL-mediated reduction in SERCA2 expression could lead to insufficient SR calcium reuptake at short pacing intervals, and thus affect the beat-to-beat stability of the calcium transient[24]. However, insufficient SR calcium reuptake would also be expected to result in a rate-dependent elevation of cytosolic calcium concentration at baseline, a phenomenon that was observed in control conditions but not with high extracellular LDL (see **Figure 3**). This observation is likely due to the concomitant LDL-mediated reduction in RyR2 and IP3R expression, which together with a reduced SERCA level is expected to cause a reduction in calcium cycling on a beat-to-beat basis. Additionally, LDL dose-dependently impaired propagation of the calcium transient across the myocyte culture at short pacing intervals, suggesting a reduction in excitability[25] and/or a reduction in the communication between the cultured cardiomyocytes[26], [27].

### LDL-mediated reduction of connexin-40 expression and signal propagation

The observation that the Cx40 protein expression is dose dependently reduced as the LDL-induced accumulation of intracellular CE increases could account for the observed LDL-mediated reduction in the propagation velocity of the calcium signal[26]. Moreover, the observation that intracellular CE derived from LDL plays a significant role on Cx40 reduction is in accordance with previous work in aortic endothelium where the Cx40 expression was found strongly reduced in a hyperlipidemic apoE knockout mice[28]. In contrast to the downregulatory effect of LDL on Cx40 expression, we did not observe any significant effects of LDL on CX43 expression in HL-1 cardiomyocytes, suggesting that Cx40 and Cx43 are differentially regulated by LDL in atrial cardiomyocytes. It should also be kept in mind that alterations in Cx43 phosphorylation have been reported to cause conduction abnormalities in the context of congestive heart failure[29], and we cannot rule out that changes in Cx43 phosphorylation could also contribute to the observed reduction of the propagation velocity. Similarly, changes in the activity of Na^+^ or K^+^-channels could also modify the propagation of the electrical signal, and we cannot rule out that LDL-mediated lipid accumulation also modulates the propagation velocity by changing Na^+^ or K^+^-channel expression or activity

On the other hand, previous studies in mouse and goat models have reported that lack of Cx40 increases atrial vulnerability to arrhythmia[30], [31], suggesting that the LDL-mediated reduction in Cx40-expression reported here might increase the vulnerability to atrial arrhythmia. In line with this notion, a marked increase in intracellular lipid droplets has been associated to the disruption of intercellular junctions in the biopsied myocardium from a patient with arrhythmogenic right ventricular cardiomyopathy[32].

### Considerations on the model

Although our findings cannot be directly extrapolated to clinical conditions, cultured atrial HL-1 myocytes retain fundamental features of the native cardiomyocyte such as intracellular calcium transients and contraction in response to electrical field stimulation as well as propagation of the calcium signal through the cultured adult cardiomyocytes[20], [21]. Moreover, this *in vitro* model may be useful to investigate effect of high LDL levels on intracellular lipid accumulation and calcium handling in cardiomyocytes, and appropriate for studying concurrent effects of LDL on the expression of calcium handling proteins, on the intracellular calcium transient, and on its propagation through the multicellular myocyte preparation.

In summary, our results highlight LDL-cholesterol as a potential contributor to reductions of the calcium transient amplitude and its propagation in cardiomyocytes by suppressing expression of Cx40 and the calcium handling proteins SERCA2, RyR2, IP2-RI and IP3-RII.
